# Accurate training of the Cox proportional hazards model on vertically-partitioned data while preserving privacy

**DOI:** 10.1186/s12911-022-01771-3

**Published:** 2022-02-24

**Authors:** Bart Kamphorst, Thomas Rooijakkers, Thijs Veugen, Matteo Cellamare, Daan Knoors

**Affiliations:** 1 Cyber Security and Robustness, Netherlands Organisation for Applied Scientific Research, The Hague, The Netherlands; 2 Research and Development, Netherlands Comprehensive Cancer Organisation, Eindhoven, The Netherlands; 3 Cryptology, Centrum Wiskunde and Informatica, Amsterdam, The Netherlands

**Keywords:** Cox proportional hazard, Secure multi-party computation, Vertically-partitioned data

## Abstract

**Background:**

Analysing distributed medical data is challenging because of data sensitivity and various regulations to access and combine data. Some privacy-preserving methods are known for analyzing horizontally-partitioned data, where different organisations have similar data on disjoint sets of people. Technically more challenging is the case of vertically-partitioned data, dealing with data on overlapping sets of people. We use an emerging technology based on cryptographic techniques called secure multi-party computation (MPC), and apply it to perform privacy-preserving survival analysis on vertically-distributed data by means of the Cox proportional hazards (CPH) model. Both MPC and CPH are explained.

**Methods:**

We use a Newton-Raphson solver to securely train the CPH model with MPC, jointly with all data holders, without revealing any sensitive data. In order to securely compute the log-partial likelihood in each iteration, we run into several technical challenges to preserve the efficiency and security of our solution. To tackle these technical challenges, we generalize a cryptographic protocol for securely computing the inverse of the Hessian matrix and develop a new method for securely computing exponentiations. A theoretical complexity estimate is given to get insight into the computational and communication effort that is needed.

**Results:**

Our secure solution is implemented in a setting with three different machines, each presenting a different data holder, which can communicate through the internet. The MPyC platform is used for implementing this privacy-preserving solution to obtain the CPH model. We test the accuracy and computation time of our methods on three standard benchmark survival datasets. We identify future work to make our solution more efficient.

**Conclusions:**

Our secure solution is comparable with the standard, non-secure solver in terms of accuracy and convergence speed. The computation time is considerably larger, although the theoretical complexity is still cubic in the number of covariates and quadratic in the number of subjects. We conclude that this is a promising way of performing parametric survival analysis on vertically-distributed medical data, while realising high level of security and privacy.

## Background

In biomedical research, linking data from multiple sources can make analyses more robust and allows studies to take into account additional information. These combined datasets might reveal patterns that the data in isolation cannot. For example, after combining data from cancer registries and general practices, additional information of the patient can be included in survival studies, e.g. comorbidities, alcohol consumption, or prescribed drugs. By doing so, individuals’ clinical and demographic characteristics can be taken into account when developing risk prediction models. A more individualized approach to survival analysis will lead to more accurate information for patients and clinicians to support their decision-making [1]. Similarly, in another study, where cancer registry data was combined with pharmaceutical data, aspirin use after the diagnosis of colon cancer was associated with increased overall survival [2]. Evidently, combining data can lead to discoveries with profound clinical implications.

Due to the sensitive nature of medical data, however, it is challenging for organisations to share and combine data. Legal frameworks like the General Data Protection Regulation^1^(GDPR) and the Health Insurance Portability and Accountability Act^2^ (HIPAA) further restrict the usage and exchange of medical data. This challenge is holding back research and our ability to reflect on the care and prevention of diseases. To overcome this challenge while respecting such regulation, new methods are needed to enable research on distributed data while protecting patient sensitive information.

We distinguish two types of distributed data: horizontally-partitioned and vertically-partitioned. When data is horizontally-partitioned, each data holder stores identical items but of different patients, e.g. national cancer registries. Advances in machine learning have led to methods for federated learning, which are able to train models on horizontally-distributed data. With federated learning, statistical techniques are broken down in multiple parts to run on distributed datasets. Only aggregated statistics are shared between parties and the final result is equivalent to the same analysis being performed on the combined dataset. Federated learning is already used in several clinical studies with data from multiple institutions [3, 4]. In general, however, it is hard to determine whether the statistics that are shared do not reveal more than intended. In some cases these can be susceptible to reconstruction attacks [5].

When data is vertically-partitioned, each data holder manages different items but about the same sets of patients. While federated learning might work in some vertical cases, it is often a lot more challenging since correlations between covariates are locally unknown. Instead, another promising technology in the form of secure multi-party computation (MPC) is able to deal with this scenario while achieving high levels of security. This technique is discussed in more detail below.

In this article we focus on the latter scenario and propose a privacy-preserving version of the Cox proportional hazards (CPH) [6] model for vertically-partitioned data. The primary audience of this paper are application-driven researchers that are interested in privacy-preserving survival analyses, such as clinical data scientists and applied cryptologists. Together, these disciplines are able to fully leverage the information that is hidden in distributed, sensitive data sources, improve our understanding of complex diseases and therewith contribute to improved treatment and well-being of patients. Although the motivation of the presented research is clinical oncology, the results generalize to many other types of biomedical, clinical studies.

### Cox proportional hazards

The Cox proportional hazards model is a multivariate regression model commonly used in medical research for investigating the impact of one or more covariates on the survival probabilities of subjects. It is a widely-recognized tool in survival analysis of a particular group of subjects ${\mathcal {I}}$ participating in an ‘experiment’, and at any time they either (1) continue to ‘live’ in the experiment, (2) ‘fail’ in the experiment, or (3) decide to no longer participate in the experiment, and hence become ‘censored’. The CPH model explicitly takes this last group of subjects into account, as their data is valuable even if they did not finish the experiment.

For a subject $I\in {\mathcal {I}}$ with *p* covariates $\varvec{Z}^I = (Z_1^I, \ldots , Z_p^I)$, the CPH model assumes that the *hazard function* $\lambda (t|\varvec{Z}^I; \varvec{\beta })$ can be expressed as

1 $$\begin{aligned} \lambda (t|\varvec{Z}^I; \varvec{\beta }) = \lambda _0(t)\exp \Big [\beta _1 Z^I_1+\beta _2 Z^I_2 + \cdots + \beta _p Z^I_p\Big ] \end{aligned}$$

where $\lambda _0(t)$ is the *baseline hazard function* that reflects the underlying hazard for subjects with all covariates equal to zero (also-called ‘reference group’) and $\varvec{\beta } = (\beta _1, \ldots , \beta _p)$ is the trained model vector that assigns a weight to every covariate that corresponds to its impact on the hazard of a subject.

An important aspect of Cox regression model is that the relative risk of two events is constant over time. A consequence of this property is that, if we write the log of the hazard ratio for subject *I* to the baseline,

$$\begin{aligned} \log \left[ \frac{\lambda _I(t)}{\lambda _0(t)}\right] =\beta _1 Z^I_1+\beta _2 Z^I_2 + \cdots + \beta _p Z^I_p, \end{aligned}$$

then the CPH model simplifies to a linear model for the log of the hazard ratio. The main advantage of this family of regression models is that we can estimate the parameters $\varvec{\beta }$ without having to estimate $\lambda _0(t)$ and hence without assuming any particular parametric model for the baseline hazard function; in particular, the model is semi-parametric. In this article, we train the CPH model using Breslow’s approximation of the partial likelihood function [7].

### Secure multi-party computation

MPC is a cryptographic technique that enables multiple parties, each having private inputs, to jointly evaluate a function on their data, without revealing their data to each other. Starting in 1982 with Yao [8], the field has become more mature in the final decade with a so-called share-compute-reveal approach. In this approach, the secret data of every party is distributed in *shares* that independently do not reveal any information about the secret data, but can together be used to reconstruct the secret data. These shares are distributed over the parties, who are then able to perform computations with the shares that correspond to the operations that one would like to perform on the original secret data. When the final, manipulated shares are combined, the parties obtain the result of the computation as if it were performed in the traditional, non-cryptographic way. In particular, it is possible to add, multiply and compare secret values of different parties without revealing their data.

The field has further progressed by e.g. adding a preprocessing phase for speed-up, leading to efficient MPC platforms like SPDZ [9] and MASCOT [10]. We use MPyC [11] for our implementations, a Python based framework based on Shamir secret sharing [12]. MPyC uses the semi-honest security model, meaning that parties might be curious to learn sensitive data of other parties, but are not able to derive this data as long as they follow the rules of the cryptographic protocol. This assumption resembles the healthcare setting where the main goal of collaboration is to obtain new medical insights.

More formally, we assume that at least half of the parties do not collude with others (e.g. share information) to deduce information. In order to protect the security and integrity of the exchanged information, we assume that the communication channels have been end-to-end encrypted. In this setting, the only way for parties to deduce each other’s sensitive information, is from (1) their own input data and (2) the received output of the computation, which from a security perspective is the best we can achieve.

### Related work

In 2016, Shi et al. [13] presented a solution for grid logistic regression on horizontally-partitioned data. While using MPC they ran into problems of securely inverting the Hessian matrix and computing natural exponentiation, but they were able to find workarounds. As our situation is more complex, due to increased algorithm complexity and different data partitioning, we had to find different solutions for these challenges, which are described in “Secure exponentiation protocol” and “Matrix inverse protocol” sections respectively.

Several publications describe approaches for privacy-preserving Cox regression. The works by Yu et al. [14] and Lu et al. [15] consider horizontally-partitioned data, whereas the recent work of Dai et al. [16] assumes vertically-partitioned data. The work by Domadiya and Rao [17] also considers vertically-partitioned healthcare data, for which they present a privacy-preserving association rule mining technique.

Yu et al. preserve privacy by mapping the data to a lower dimensional space [14]. They construct their affine, sparse mapping by solving a linear program that optimizes the map in such a way that certain properties are maintained (e.g. ordering imposed by survival time, covariate selection) and thereby improve on earlier works that use random mappings. The Cox model is publicly trained on the lower-dimensional data and achieves near-optimal performance.

Lu et al. design and implement a Web service, WebDISCO, for joint training of a Cox regression model [15]. Based on federated learning ideology, they achieve privacy-preservation by sharing aggregated information only instead of individual data records. The obtained model is mathematically equivalent to a model that is trained directly on the joint data.

Dai et al. consider vertically-partitioned data and leverage the alternating direction method of multipliers (ADMM) [18] to directly train the model to its optimum^3^ [16]. Note that the ADMM method itself is iterative. The authors present their work in a client-server setting where each client only transmits aggregated intermediary results to the server in each iteration. The server performs heavier computations than the client. The subject-level data never leaves the client’s organization, although all parties must know which subjects experienced an event (not the event time). The final model is equivalent to the model that is trained directly on the joint data.

Our work also assumes vertically-partitioned data, but otherwise follows a different approach from Dai et al. [16]. Firstly, instead of a direct approach, we leverage the Newton–Raphson method for iterative training of the CPH model. Secondly, we perform all computations in the encrypted domain using secure multi-party computation instead of computations in the plain where privacy is preserved through aggregation. Aggregation may provide a solid preservation of privacy; however, in practice it is hard to make this precise and obtain mathematical guarantees on the security that is provided.

Our contributions are the following:

- A novel protocol for training a privacy-preserving CPH model in the encrypted domain. The model is trained in an iterative fashion using the Newton–Raphson method for optimizing Breslow’s partial likelihood function.
- Fundamental and widely-applicable protocols for computing exponentiations in the secure domain. That is, we securely compute $a^x$ for known $a>0$ and encrypted *x*.
- A new protocol for securely inverting a non-integer matrix. We use a known approach for integer matrices, and adjust it to our needs.
- A recursive approach for accurately computing the gradients without using floating point arithmetic.

Privacy-preservation of input data during computation is an important aspect of privacy-preserving machine learning. However, preserving privacy during computation by means of aggregation or encryption does not prevent a malicious user to deduce sensitive information from the output of the computation. Although we did not look into this aspect in our work, we do want to mention some works that consider this aspect. O’Keefe et al. [19] describe several methods for what they call “confidentialising” the CPH output. For example, they suggest that using a random 95% of the training data, robust estimators and rounded or binned outputs can reduce the information leakage of the CPH output while preserving the most important characteristics. Although some of the techniques seem to improve privacy preservation, one should note that no mathematical guarantees of the effectiveness of the presented techniques are presented.

Another approach is persued by Nguyên and Hui [20] and Nguyên [21], who design differentially private methods for generalized linear models and the CPH model. Differential privacy is a mathematical framework ensuring that an adversary is not able to deduce the exact private information of a targeted subject from the trained model [22]. This is achieved by adding noise to the data, the penalty function or the trained model and usually result in an accuracy-privacy trade-off. The work of Nguyên [21] does not consider distributed data. In contrast, we consider distributed data and no noise is added anywhere in the process. Both works may yield interesting and partially orthogonal complements to our work.

## Methods

In this section, we train a CPH model on confidential, vertically-partitioned data. We assume that (at least) two parties know several complementary covariates of the same set of subjects and wish to collaboratively train a CPH model without ever revealing the personal data in their possession to each other. Their goal is achieved by means of MPC protocols.

This section is structured as follows. We first elaborate on the distribution of data over the participating parties. Subsequently, we introduce the partial likelihood of CPH model and explain how it can be optimized using a Newton–Raphson solver. Finally, we introduce the privacy-preserving protocols that train the CPH model in the encrypted domain, thereby greatly enhancing the subjects’ privacy. Two generic building blocks, secure exponentiation and secure matrix inverse, are described in separate sections to highlight their independence of the secure CPH protocol.

A list of symbols used throughout this section is presented in Table 1.

**Table 1:** Overview of symbols

Symbol	Definition
*m*	Number of parties involved in the MPC protocol
*N*	Secret-sharing modulus
*n*	Number of subjects
${\mathcal {I}}$	Set of all subjects
*J*	Number of distinct event times
${\mathcal {D}}_j$	Set of subjects that experience an event at time $t_j$
$d_j$	Number of subjects that experience an event at time $t_j$; $|{\mathcal {D}}_j|$
${\mathcal {R}}_j$	Set of subjects at risk (alive and uncensored) at time $t_j$
$L(\cdot )$	Log-likelihood function for CPH model
*p*	Number of covariates
$\varvec{Z}$	*p*-Dimensional vector of explanatory covariates
$\varvec{\beta }$	*p*-Dimensional vector of model parameters
$\varvec{Z}^I$	Realisation of the *p*-dimensional covariate vector $\varvec{Z}$ for subject *I*

### Participating parties and their data

The setting of this article is that $m\ge 2$ parties wish to jointly train a CPH model on their sensitive, vertically-partitioned data. One of the parties provides the event time $X^I$, censoring information $\delta ^I$ and possibly a subset of covariates $Z^I_1, \ldots , Z^I_{p_1'}$. Every other party *i* provides complementary covariates $Z^I_{p_{i-1}'+1}, \ldots , Z^I_{p_i'}$, $p_m'=p$ for the same subjects *I*. Together, the parties thus have a richer understanding of every subject in their shared population. Note that this is different from a horizontal partitioning, where every party has the same covariates of different populations.

In our implementation we assume that, if there are only two data-owning parties (e.g. there is no third party who also contributes data to the computation), then there exists a helper party who is trusted to the extend of truthfully evaluating the protocol and not colluding with others. However, no sensitive data is revealed to the helper and as such the required level of trust in the helper is lower than what would be required for a (traditional) trusted third party that obtains all the data. The additional helper participates in the protocol for security and efficiency reasons only; all secure protocols can be implemented in a suitable two-party MPC framework.

In practice, it is unlikely that the datasets of all players contain precisely the same subjects. Moreover, it is not quite certain that the subjects are ordered in the same manner. It is possible to start from this setting and then progress to a situation where all parties made a (secure) selection such that both datasets contain the same subjects and in the same order. Depending on the type of identifiers used, this can be achieved in traditional ways, or by using cryptographic protocols [23]. Instead, in this article we assume that the intersecting and aligning of databases has already been performed in such a pre-processing phase, which allows us to focus on the secure implementation of the CPH model.

### Optimizing Breslow’s approximation

The estimation procedure for the CPH model uses a partial likelihood approach that produces estimates for $\beta$ without involving $\lambda _0(t)$. The estimate depends on observations $(X^I,\delta ^I,\varvec{Z}^I)$ for every subject *I* that participates in the experiment, where $X^I$ is the censored failure time random variable, $\delta ^I$ the failure-censor indicator and $\varvec{Z}^I$ the set of covariates. In particular, $\delta ^I = 1$ if the subject failed at time $X^I$ whereas $\delta ^I = 0$ if the subject got censored at time $X^I$, presumably because the subject stopped participating in the experiment while being alive.

We consider Breslow’s approximation [7] of the partial likelihood, which allows for tied event times $X_I=X_J$. The approximation groups subjects according to their censored event time, so let $t_1\le t_2 \le \cdots \le t_J$ denote the unique elements of the set $\{X^I: \delta ^I=1\}$. That is, the first time that there were actual failures among all subjects was $t_1$, the second such time was $t_2$, etc. We refer to these times as the *distinct event times*. We define ${\mathcal {D}}_j:= \{I: X^I = t_j, \delta ^I = 1\}$ as the set of subjects that experienced failure at time $t_j$ and let ${\mathcal {R}}_j:= \{I: X^I \ge t_j\}$ denote the set of subjects that were at risk at time $t_j$. Finally, the number of actual failures at time $t_j$ is denoted by $d_j:=|{\mathcal {D}}_j|$. Breslow’s approximation of the partial likelihood is given by

2 $$\begin{aligned} L(\varvec{\beta }) = \prod _{j=1}^J \frac{\prod _{I\in {\mathcal {D}}_j} e^{\varvec{\beta }^T\varvec{Z^I}}}{\left( \sum _{I\in {\mathcal {R}}_j} e^{\varvec{\beta }^T\varvec{Z^I}}\right) ^{d_j}}. \end{aligned}$$

Cox recommended to treat the partial likelihood as a regular likelihood for making inferences about $\varvec{\beta }$, in the presence of the nuisance parameter $\lambda _0(\cdot )$. Therefore, let us consider the log-partial likelihood

3 $$\begin{aligned} l(\varvec{\beta })&= \log (L(\beta )) \\&= \sum _{j=1}^J\left\{ \varvec{\beta }^T\sum _{I\in {\mathcal {D}}_j} \varvec{Z}^I - d_j \log \left[ \sum _{I\in {\mathcal {R}}_j} e^{\varvec{\beta }^T\varvec{Z}^I}\right] \right\} . \end{aligned}$$

We aim to optimize the log-likelihood by applying the iterative Newton–Raphson method;

4 $$\begin{aligned} \varvec{\beta }^{t+1}=\varvec{\beta }^t - \left( \nabla ^2 l(\varvec{\beta }^t)\right) ^{-1}\nabla l(\varvec{\beta }^t). \end{aligned}$$

Here, $\nabla l(\varvec{\beta }) = \left( \frac{\partial l}{\partial \beta _1}(\varvec{\beta }), \ldots , \frac{\partial l}{\partial \beta _p}(\varvec{\beta })\right)$ and $\nabla ^2 l(\varvec{\beta }) = \left( \frac{\partial ^2 l}{\partial \beta _r \partial \beta _s}(\varvec{\beta })\right) _{r,s\in \{1, \ldots , p\}}$ are the gradient and the Hessian matrix of $l(\varvec{\beta })$, respectively. Their elements are given by

5 $$\begin{aligned} \frac{\partial l}{\partial \beta _r}(\varvec{\beta }) = \sum _{j=1}^J \left\{ \sum _{I\in {\mathcal {D}}_j} Z_r^I - d_j \frac{\sum _{I\in {\mathcal {R}}_j} Z_r^I e^{\varvec{\beta }^T \varvec{Z}^I}}{\sum _{I\in {\mathcal {R}}_j} e^{\varvec{\beta }^T \varvec{Z}^I}}\right\} \end{aligned}$$

and

6 $$\begin{aligned}&\frac{\partial ^2 l}{\partial \beta _r \partial \beta _s}(\varvec{\beta }) =  -\sum _{j=1}^J d_j \left\{ \frac{\sum _{I\in {\mathcal {R}}_j} Z_r^I Z_s^I e^{\varvec{\beta }^T \varvec{Z}^I}}{\sum _{I\in {\mathcal {R}}_j} e^{\varvec{\beta }^T \varvec{Z}^I}} \right. \\&\quad - \left. \frac{\sum _{I\in {\mathcal {R}}_j} Z_r^I e^{\varvec{\beta }^T \varvec{Z}^I}}{\sum _{I\in {\mathcal {R}}_j} e^{\varvec{\beta }^T \varvec{Z}^I}} \times \frac{\sum _{I\in {\mathcal {R}}_j} Z_s^I e^{\varvec{\beta }^T \varvec{Z}^I}}{\sum _{I\in {\mathcal {R}}_j} e^{\varvec{\beta }^T \varvec{Z}^I}}\right\} \end{aligned}$$

for every $r, s \in \{1, \ldots , p\}$. Alternative representations of and methods for training CPH model are discussed by [16, 24–26].

### Secure CPH protocol

This section presents a secure version of the Newton–Raphson solver that was described above for training the CPH model. As we elaborate on every step of the protocol, we pay special attention to the limitations of fixed-point arithmetic and finite fields that motivated us into designing the most interesting protocol steps.

#### Overview secure CPH protocol

We now provide the blueprint of our secure *m*-party implementation of the CPH protocol. The secure protocol can be implemented in any linear secret-sharing platform that tolerates a dishonest minority of up to *t* passively corrupt parties (e.g. $0\le t < m/2$). The plaintext modulus is denoted by *N* and secrets are denoted by [.]. The statistical security parameter is denoted by $\sigma$. We assume that the platform supports fixed-point arithmetic rather than floating-point arithmetic. When a secret value *x* is split into shared for distribution and computation, we say that *x* is *secret-shared*; contrastingly, information that is *shared* has not been encrypted unless mentioned otherwise.

The idea is to secret-share the data $\varvec{Z}^I$ and perform some one-time manipulations. From the secret-shared data, we compute secret-shared model parameters $\beta _r$, $1\le r\le p$ which are updated in each iteration according to Eq. (4) without ever revealing them. Only when the model has converged do we combine the shares to reveal the desired output. This iterative process is broken down into several steps, which we present in Protocol 1. Before these steps are explained in more detail in the following subsections, we evaluate the information that is shared between the participating parties in unencrypted form.
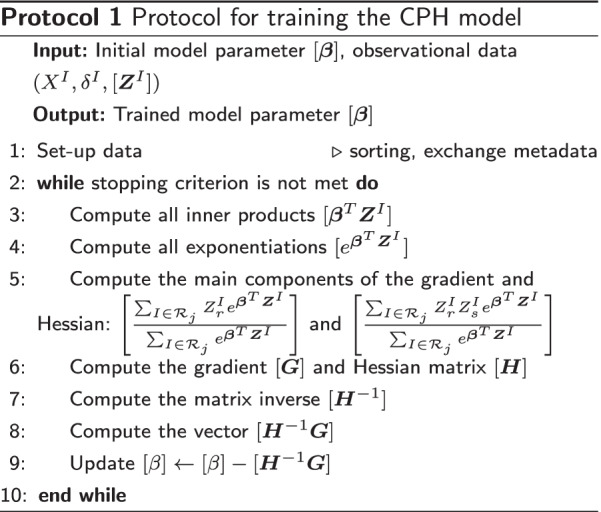


#### Shared information

During the Newton–Raphson iterations, the vector $\varvec{\beta }$ converges to a final value. As intermediate values of $\varvec{\beta }$ might reveal some information about other parties’ input data, the parties are allowed to see the converged vector only. The MPC technology assures that, apart from the actual number of iterations and the output of the model, the only other information that is learned by the involved parties is:

- The number of distinct event times, but not the timestamp of these events.
- The number of events and censorings per distinct event time index, but not the corresponding subjects.
- The total number of subjects.
- The number of covariates per party.
- Scaling factor per covariate (optional).

While this information could remain hidden, this would require a lot more effort and computational overhead. Because this information does not appear to reveal any sensitive information with respect to the individual subjects, we assume that this can be revealed to the involved parties.

#### Data set-up

After secret-sharing the data $\varvec{Z}^I$, we perform some one-time manipulations that rely on the data distribution. In particular, we sort the rows of the joint, secret-shared database such that they are sorted in ascending order of event time and exchange some metadata. This is facilitated by the fact that one of the parties, say party 1, provides both the event time period $X^I$ and the censoring information $\delta ^I$ of every subject *I*.

Securely sorting the rows of the joint database such that they are sorted in ascending order of event time corresponds to multiplication of the joint database by a suitable permutation matrix. Party 1 can locally produce the permutation matrix that corresponds to this change from the locally-available event times and secret-share it. The parties can then jointly perform the permutation.

Additionally, party 1 shares some metadata in unencrypted form. First, party 1 shares a list that, for every distinct event time, indicates the indices of the rows that correspond to subjects that experienced an event. Second, party 1 shares a list that, for every distinct event time, indicates the indices of the lists that correspond to subjects at risk. After this phase, all parties are able to select those rows that correspond to patients in ${\mathcal {D}}_j$ and ${\mathcal {R}}_j$ without actually knowing which patients they selected or the attributes of these patients. The information now shared also reveals $d_i$.

The reason for sharing this information is that it is non-sensitive, yet sharing this information significantly simplifies evaluation of the non-trivial summations in expressions (5) and (6).

#### Secure inner products

The first step of Protocol 1 (in line 3) is to securely compute the inner products $[\varvec{\beta }^T\varvec{Z}^I]$. Since secret sharing doesn’t allow for local multiplication of privately known numbers (the $Z^I_r$), the obvious solution is that each party *k* generates secret sharings of $Z^I_r$ for each subject *I* and every covariate *r* known to party *k*. Then the inner product $[\varvec{\beta }^T\varvec{Z}^I]$ can be computed by *p* secure multiplications, and $m-1$ (local) additions. Note that secure multiplications require a round of communication between the parties.

#### Secure exponentiations

The second step of Protocol 1 (in line 4) is to compute $[e^x]$, given the encrypted number [*x*] (where [*x*] e.g. resembles the secure inner products computed before). The sub-protocol for doing this is described in “Secure exponentiation protocol’ section as it is of independent interest. At this point we only note that the sub-protocol suffers from the fixed-point representation of numbers, implying that $e^x$ only fits within the finite field for a very limited range of *x*.

Assume that secure numbers are represented with 32 bits and a fixed-point encoding that uses the first 16 bits to encode the sign bit and integer part. Then the range of secure numbers is $[-2^{15}, 2^{15}]$ with a granularity of $2^{-16}$. For $e^x$ to fit in this range, we can only allow for $x\in [-12,12]$. Our implementation securely truncates [*x*] such that it falls within this range. The feasible domain can be enlarged by using more bits in the representation of secure numbers; however, this will only marginally increase the feasible domain due to the exponential growth whereas the computation time of the protocol is significantly increased. As such, we designed the remaining steps of the protocol to cope with the limited feasible domain of the secure exponentiation.

#### Secure gradient and Hessian matrix

The expressions for the gradient and Hessian matrix, expressions (5) and (6), involve many components that are non-trivial to evaluate in the encrypted domain. We present our discussion in terms of the gradient since the computation of the Hessian can be done in a similar fashion.

Due to the data set-up phase, all parties know which rows in the joint secret-shared database correspond to ${\mathcal {D}}_j$ and ${\mathcal {R}}_j$ for all $j\in \{1, \ldots , J\}$. Additionally, the multiplication by $d_j$ can be performed by every party locally as their values have also been exchanged. We thus need to show how $\frac{\sum _{I\in {\mathcal {R}}_j} Z_r^I \exp [\varvec{\beta }^T \varvec{Z}^I]}{\sum _{I\in {\mathcal {R}}_j} \exp [\varvec{\beta }^T \varvec{Z}^I]}$ can be evaluated securely and accurately. This is especially challenging due to the limited feasible domain of the secure exponentiations that was discussed before. We will elaborate on our approach to evaluate the fraction accurately.

For the remainder of this section, without loss of generality, we assume that the subjects *I*, which are sorted according to ascending event time, are numbered from 1 to *n*. Let $r_j$ denote the number of subjects at risk at the *j*-th distinct event time. Then, by choice of our ordering and numbering, we find that ${\mathcal {R}}_j = \{n-r_j+1, n-r_j+2, \ldots , n\}$. We denote

7 $$\begin{aligned} G_r^k := \frac{Z_r^k e^{\beta ^T\cdot \varvec{Z}^k} + \cdots + Z_r^n e^{\varvec{\beta }^T\cdot \varvec{Z}^n}}{e^{\varvec{\beta }^T\cdot \varvec{Z}^k} + \cdots + e^{\varvec{\beta }^T \cdot \varvec{Z}^n}} \end{aligned}$$

and are thus primarily interested in the values of $G_r^k$ for $k = n-r_j+1$, $j=1,\ldots ,J$, as these $G_r^k$ correspond to the $\frac{\sum _{I\in {\mathcal {R}}_j} Z_r^I \exp [\varvec{\beta }^T \varvec{Z}^I]}{\sum _{I\in {\mathcal {R}}_j} \exp [\varvec{\beta }^T \varvec{Z}^I]}$.

Due to the limited feasible domain of the secure exponentiation and the corresponding truncation, it is highly likely that direct, secure evaluations of expression (7) result in inaccurate values. Fortunately, one may see that $G_r^k$ is a weighted average of $Z_r^i$, meaning that it is the ratio between weights that impacts the final value rather than the absolute weights. In particular, one may write

8 $$\begin{aligned} G_r^k&= \frac{Z_r^k}{1 + e^{\varvec{\beta }^T\cdot \varvec{Z}^{k+1} - \beta ^T\cdot \varvec{Z}^k} + \cdots + e^{\varvec{\beta }^T \cdot \varvec{Z}^n - \beta ^T\cdot \varvec{Z}^k}} \\&\quad + \cdots + \\&\frac{Z_r^n}{e^{\varvec{\beta }^T\cdot \varvec{Z}^k - \beta ^T\cdot \varvec{Z}^n} + \cdots + e^{\varvec{\beta }^T \cdot \varvec{Z}^{n-1} - \beta ^T\cdot \varvec{Z}^n} + 1} \\&=: \eta ^k_k Z_r^k + \cdots + \eta ^k_n Z_r^n, \end{aligned}$$

where $\eta ^k_i\in (0,1]$, to see that we only truly care about the ratio between the various exponentiated inner products. This seems to solve the challenge posed by the limited range of exponentiation; one could directly compute the $G_r^k$ from the approximated $\eta ^k_i$ for all relevant *k*. However, $G_r^k$ needs to be computed for $k = n-r_j+1$, $j=1,\ldots ,J$ and the *j*-th evaluation requires the computation of $r_j$ values of $\eta ^k_i$. As we expect $J = O(n)$ this implies secure evaluation of roughly $\frac{1}{2}n^2$ reciprocals per iteration of the Newton–Raphson procedure, which is expensive. This observation motivated the following alternative, recursive evaluation of relation (8):

9 $$\begin{aligned} G_r^n&= Z_r^n \ \\ G_r^{k-1}&= G_r^k + \theta ^{k-1} \cdot (Z_r^{k-1} - G_r^k), \quad k=2,\ldots ,n \end{aligned}$$

where

10 $$\begin{aligned} \theta ^{k-1}&:= \frac{1}{1 + e^{\varvec{\beta }^T\cdot \varvec{Z}^k - \beta ^T\cdot \varvec{Z}^{k-1}} + \cdots + e^{\varvec{\beta }^T \cdot \varvec{Z}^n-\beta ^T\cdot \varvec{Z}^{k-1}}} \end{aligned}$$

is equal to $\eta _k^k$ above and assumes values in (0, 1). This recursive approach requires *n* secure evaluations of a reciprocal per iteration of the Newton–Raphson procedure.

In the final expressions it becomes clear that there is no need to compute $e^x$ with high accuracy when *x* is not close to 0. If *x* is negative and large, then the contribution of that term to $\theta$ is negligible and can be approximated with zero. If *x* is positive and large, then the contribution of that term to $\theta$ indicates that $\theta$ will be close to zero. The fact that $\theta$ is close to zero may be more important than how close it is exactly (e.g. $10^{-5}$ or $10^{-10}$).

The described protocol for computing $G_r^k$ is iterative, meaning that inaccuracies in an early stage may propagate throughout all later iterations. However, our experiments show that the recursive approach yields sufficiently accurate results for our purposes.

#### Updating the model parameters

The model parameters $\beta$ are updated as in Eq. (4). The main challenge is, given the scaled Hessian matrix and gradient vector, to compute the inverse of the $p \times p$ Hessian matrix $\varvec{H}$ in the encrypted domain. Once we have computed the inverse Hessian matrix, the remaining matrix vector product is simply a number of secure inner products. The matrix inverse protocol is described in “Matrix inverse protocol” section.

#### Stopping criterion

The protocol keeps iterating until one of two conditions is met: it either completed a pre-defined maximum number of iterations or the model has converged. We say that the model is converged if all elements of the absolute model update $|\varvec{\beta }^{t+1}-\varvec{\beta }^{t}|$ are smaller than a pre-defined convergence threshold $\tau$. Since the absolute model update is secret-shared, we securely compute the binary output of $\max _r|\varvec{\beta }_r^{t+1}-\varvec{\beta }_r^{t}| <\tau$ and reveal this binary output to the participating parties.

### Secure exponentiation protocol

In this section we describe a protocol for computing $[a^x]$ from a public base $a\in {\mathbb {R}}_{\ge 0}$ and secret exponent [*x*]. In particular, this protocol can be used for evaluating $[e^x]$. We also present a wrapper for the secure exponentiation in case *x* cannot be guaranteed to be in the feasible domain.

The exponent *x* should be in a range such that $a^x$ can be represented in the finite field. As such, it is assumed that $x_L, x_U\in {\mathbb {R}}$ are provided such that $e^x$ can be meaningfully represented in the finite field for all $x\in [x_L, x_U]$. For example, if $a=2$ and secure numbers are represented with 32 bits, encoded as unsigned fixed points with 20 integral bits and 12 fractional bits, then $-12\le x_L < x_U\le 20$.

The solution that we describe implements separate protocols for exponentiation by an integer exponent and exponentiation by a non-integer exponent. The former protocol is exact, but is not applicable to non-integer exponents. It is complemented by the latter protocol, which is more broadly applicable at the costs of accuracy. The higher-level protocol for securely evaluating exponentiation splits the provided exponent into an integer and non-integer part and then deduces the intended result from these two protocols. The outline of the protocol is presented in Protocol 2.
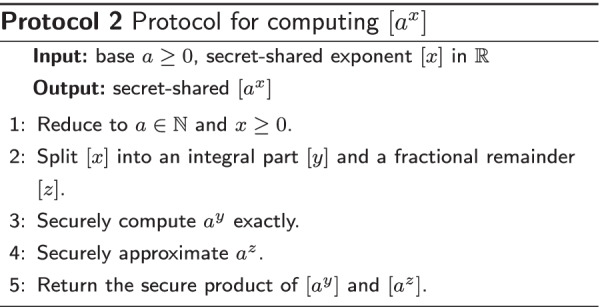


We will show that the relative error of the approximation for $a^z$ can be quantified and improved by computing a higher-order approximation polynomial. Since the final approximation for $a^x$ is the product of an exact term and term with bounded relative error, it follows that the approximation for $a^x$ of this protocol also has bounded relative error and can be tailored to the specific needs.

The above outline is identical to the approach of Thissen [27, Chapter 5]; however, our implementation differs from theirs on several points. Firstly, we use probabilistic truncation opposed to their more expensive deterministic truncation. This choice relates closely to the second difference: we utilize a MacLaurin series in our approximation of $[a^z]$, Thissen uses the polynomial $p_{1045}$ from [28] instead. The MacLaurin series is more flexible in the sense that it works for negative and positive *z* and it can be easily computed for any base *b*. Instead, $p_{1045}$ only provides guarantees on the relative error for positive *z* and requires $b=2$. This inflexibility is justified by the fact that the same accuracy can be achieved with a lower-degree polynomial approximation. Thirdly, Thissen’s protocol for computing $[2^y]$ is based on a bit decomposition of [*y*], whereas our protocol depends on additive blinding.

We now discuss the elements of our secure exponentiation in more detail.

#### Reduce input space

It suffices to have a protocol that computes $[a^x]$ from an integer base *a* and a secret-shared fixed point [*x*]. To see this, note that one can write $b^x = a^{x \log _a b}$. As such, we can just reformulate our problem of computing $[b^x]$ to computing $[a^{\tilde{x}}]$ for any integer base *a*.

Negative exponents are bothersome to work with, especially in a finite field. Instead, it is more convenient to work only with positive exponents *x*. To this end we could first securely compute the sign of *x* and denote it by $[\delta ]$, where $\delta = (x \ge 0)$. That is, $\delta =1$ if *x* is non-negative and $\delta =0$ if *x* is negative. Then we compute the exponentiation of the positive value $y = x - (1-\delta ) x_L$. Finally, we compute $e^x = \delta \cdot e^y + (1-\delta ) \cdot e^y \cdot e^{x_L}$. This solution doubles the range of acceptable *x* for a given modulus *N* at the cost of a single secure comparison.

#### Split exponent

Splitting the exponent into an integer part and a non-integer part can be done in various ways. Rounding [*x*] to the nearest integer requires an intensive secure comparison protocol, but we can use probabilistic truncation (see [29, Protocol 4.32], or [30, Protocol 2]) instead to avoid the secure comparison at a small loss of accuracy. In particular, if we let [*y*] denote the result of a probabilistic truncation of [*x*] then *y* can both be the nearest smaller or the nearest larger integer to *x*. The non-integral part [*z*] is then computed as $[x]-[y]$ and assumes a value in $(-1, 1)$.

#### Integer exponent

Assume we have *m* parties having an additive secret-sharing of *x* modulo *N*, and we would like to compute $[a^y]$, where *a* is a known integer and [*y*] is a secret-shared integer.

The outline of the subprotocol is to additively blind [*y*] with $[\sum _m r_i]$, compute the exponentiation with the resulting public value $\tilde{y}$ and correct the final result for blinding. Here, the $r_i$ are random numbers with $\sigma$ bits more than *y*. In order to produce an efficient solution we restrict the length of *y* such that $m\cdot y\cdot 2^\sigma < N$; that is, the blinded version of *y* should fit in the finite field. The subprotocol is presented in Protocol 3. Note that the value of $\tilde{y}$ obtained in step 3 has not been reduced since $0\le \tilde{y} = -y + \sum _{i=1}^m r_i \le m\cdot y\cdot 2^\sigma <N$.
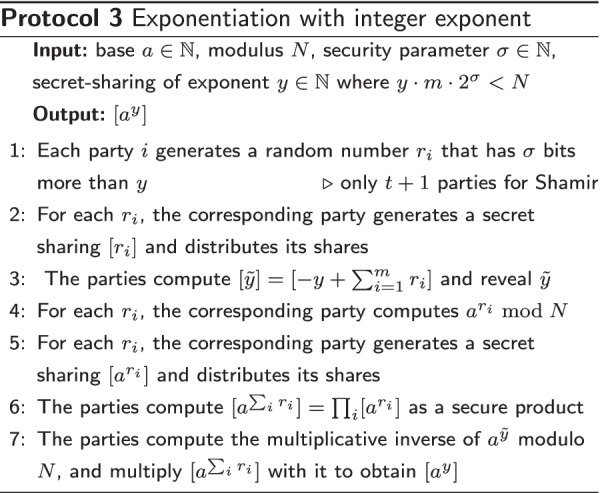


#### Non-integer exponent

We just described a protocol for computing $[a^y]$ for integer [*y*]. The standing challenge is to perform a secure exponentiation with a secret floating-point exponent *z*. We approach this challenge with a polynomial approximation of exponentiation; in particular, we base our approach on the MacLaurin series of $e^s$. This approach is justified by a short derivation that is presented in Additional file 1.
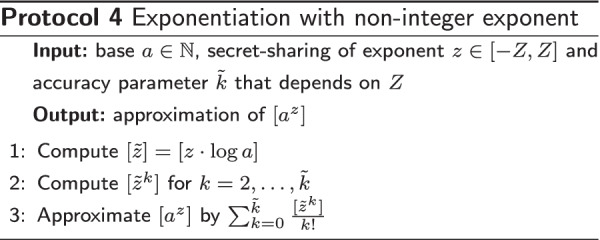


The subprotocol for exponentiation with a non-integer exponent *z* is presented in Protocol 4. In the protocol, the $\log$-function denotes the natural logarithm. For a given base *a* and a given range $[-Z, Z]$ that contains *z*, the relative error of the approximation can be made arbitrarily small by choosing an appropriate number $\tilde{k}$ of terms. For our purposes, $\tilde{k}$ typically assumes a value close to 7. Note that there is no need for expensive secure reciprocals as the reciprocals 1/*k*! can be computed in the plain.

#### Wrapper for truncation

With some extra work and three secure comparisons in total, the earlier described method for reducing the input space to $x\ge 0$ can be extended to also accept *x* outside the interval $[x_L, x_U]$ and return [*y*], where

11 $$\begin{aligned} y = \left\{ \begin{array}{ll} e^{x_U} & \text{ if } x > x_U, \\ e^x & \text{ if } x_L \le x \le x_U, \\ 0 & \text{ if } x < x_L. \end{array}\right. \end{aligned}$$

The number of secure comparisons can sometimes be reduced to two. For example, if $x_L$ and $x_U$ satisfy $x_L \ge -x_U$ then we may translate *x* to $\tilde{x}=x-(x_U+x_L)/2$, which needs to be truncated to the interval $[-(x_U-x_L)/2, (x_U-x_L)/2]$, which is symmetric around zero. One secure comparison reduces this case to truncating $|\tilde{x}|$ to $[0, (x_U-x_L)/2]$ and the second comparison is required to perform the actual truncation. The results of the secure comparisons can later be reused in order to obtain the desired result.

### Matrix inverse protocol

In this section we explain the idea of Blom et al. [31] for a matrix inversion circuit that is suitable for translation to the encrypted domain (garbled circuits, homomorphic encryption, or secret sharing), yielding an efficient solution for secure matrix inversion. Since this solution is already implemented in the MPyC library for secret-shared integers, we tweak the implementation to accept secret-shared fixed points.

We first describe the solution by Blom et al. and then discuss the changes that we made such that the protocol accepts fixed-point numbers.

#### Matrix inverse for integers

We have a *d*-by-*d* (encrypted) integer matrix $\varvec{A}$ and would like to compute its inverse (if it exists). The elements of the inverse will be rational numbers, but one can show that both the determinant $\det \varvec{A}$ and the adjugate ${\text {adj}}\varvec{A}=\varvec{A}^{-1}\det \varvec{A}$ will be integer-valued, which gives a common denominator ($\det \varvec{A}$) for the rational elements of $\varvec{A}^{-1}$. Since $\det \varvec{A}$ and ${\text {adj}}\varvec{A}$ are integer-valued, these can be separately computed, without rounding errors and within a finite field, which is very convenient in the encrypted domain.
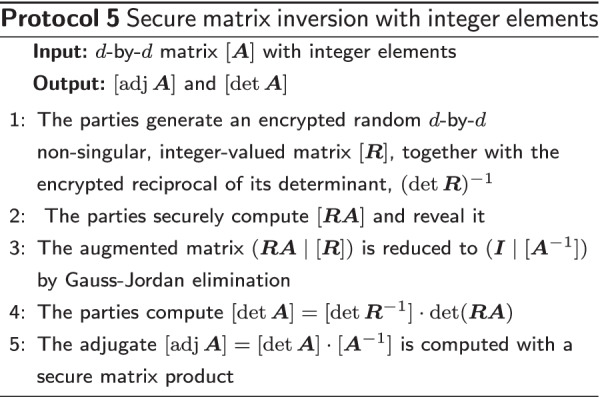


The approach of Blom et al. [31] is described in Protocol 5. Steps 1 and 3 are explained in more detail in Additional file 2. In case the determinant of $\varvec{RA}$ in step 4 is zero, we know that matrix $\varvec{A}$ is singular and stop the protocol. One should note that the term $[\varvec{A}^{-1}]$ in step 3 is the inverse of $\varvec{A}$ over ${\mathbb {Z}}_q$, where *q* is the modulus of the finite field. The inverse of $\varvec{A}$ over ${\mathbb {R}}$ is given by $(\det \varvec{A})^{-1}{\text {adj}}\varvec{A}$, where $(\det \varvec{A})^{-1}$ is the inverse of $\det \varvec{A}$ in ${\mathbb {R}}$. Blom et al. actually reveal both $[{\text {adj}} \varvec{A}]$ and $[\det \varvec{A}]$ such that the reciprocal of the determinant can be computed in the clear. For every application, one should verify that disclosing this information for gaining efficiency is acceptable.

In order to be able to properly represent the elements of the matrix inverse, the finite field should be large enough. Let $\alpha$ denote the maximal absolute value of the elements of $\varvec{A}$. One can show that $|\det \varvec{A}|\le d^{d/2}\alpha ^d$ and also derive that the maximal absolute value of the elements of ${\text {adj}}\varvec{A}$ is upper bounded by $(d-1)^{(d-1)/2}\alpha ^{d-1}$ [31]. In conclusion, computing the matrix inverse in this fashion increases the magnitude of elements from $\alpha$ to roughly $d^{d/2}\alpha ^d$. The modulus of the finite field must be chosen appropriately. Since all shares are now much larger, this blow-up puts a lot of strain on the devices’ memory. We therefore opt to convert the secret-shared elements to a larger field just before computing the matrix inverse, and convert them back to a smaller field afterwards.

#### Remarks about the MPyC implementation

Two important aspects need to be taken into consideration when using the MPyC matrix inverse: conversions between secure types and secure computation of the reciprocal of the determinant.

First, the MPyC implementation assumes that input is delivered in the MPyC SecFld format. In particular, we require secure conversions from SecInt (for integers) or SecFxp (for fixed points) to the required format. After constructing the matrix inverse, we need to perform another conversion in order to continue with the format that we started with.

The current implementation of the MPyC convert, starting with a secure number of type SecFld, involves a secure comparison and a secure modular reduction. The amount of communication required for both operations grows linearly in the number of bits *k* that is used to represent the secure number. In our case $k\approx \log _2(d^{d/2}\alpha ^d)$ grows fast with increasing matrix dimensions.

Second, as noted before, Blom et al. choose to disclose the determinant of $\varvec{A}$. However, one might not (always) wish to disclose this information, hence we opted to implement a variant where this information is kept secret. Unfortunately the secure computation of the reciprocal of $\det \varvec{A}$, particularly in the enlarged finite field, can be quite expensive.

#### Matrix inverse for fixed points

In the previous section we discussed several points of attention in the implementation of MPyC’s matrix inverse. Several additional changes need to be made in order to make the MPyC matrix inverse operational with numbers that are represented as fixed points: (1) the fixed-point numbers need to be scaled to integers and (2) this scaling needs to be corrected for later.

Assume that the chosen fixed-point representation reserves *f* bits for the fractional part of the number *x*. Then $2^f x$ is an integer. One can scale all entries in the fixed-point-valued matrix to obtain an integer-valued matrix $2^f\varvec{A}$. If we compute the matrix inverse of the scaled, integer matrix $2^f\varvec{A}$, then we obtain $2^{-f} \varvec{A}^{-1}$. Therefore, in order to correct the initial scaling, we only need to correct the final result by multiplying all elements of the inverse with $2^f$ (equivalently: scale the reciprocal of the determinant by factor $2^f$). The resulting protocol is described in Protocol 6.
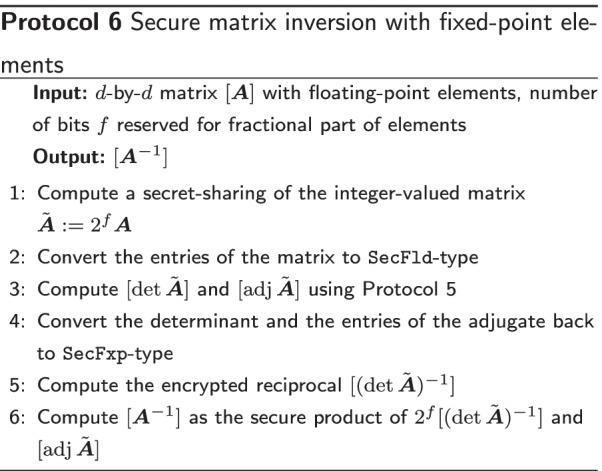


Most steps in Protocol 6 are efficient in the sense that several operations are performed in the plaintext domain rather than the encrypted domain; however, the conversion steps and the computation of the reciprocal of $\det \varvec{A}$ are quite expensive. Both components scale as a function of the number of bits $k\approx \log _2(d^{d/2}\alpha ^d)$ that are used to represent the secure numbers, which we already noted to grow fast in the dimensions of the matrix $\varvec{A}$.

### Theoretical performance

This section considers the theoretical performance of the various parts of the protocol. In particular, we present the theoretical scalability of all components in Table 2. The table gives an indication of the dependence of the performance on the number of subjects *n* and the number of covariates *p*. Some subprotocols also depend on the maximum bit-length of plaintext encodings; this dependency is not reflected in Table 2 for clarity. The next paragraphs outline the origin of the dominant terms in every subprotocol.

**Table 2:** Big-O complexity of our (sub)protocols, implemented in the MPyC framework

Building block	Invocations	Rounds
Pre-processing (one-time)	$O(n^2 + np^2)$	*O*(1)
Secure exponentiation	$O(\tilde{k}n^2)$	$O(\tilde{k})$
Computing $\varvec{G}$ and $\varvec{H}$	$O(n+Jp^2)$	*O*(*n*)
Secure matrix inverse	$O(p^3 \log p)$	$O(\log p)$
Update $\beta$	*O*(*p*)	*O*(1)
Checking convergence criterion	*O*(*p*)	*O*(1)
Secure CPH	$O(\tilde{k}n^2 + np^2 + p^3\log p)$	$O(\tilde{k} + n + \log p)$

Costs are per iteration unless stated otherwise. An invocation is the amount of data send by each party in a multiplication protocol, which also highly correlates with the number of operations that need to be performed locally by each player. The number of communication rounds is estimated for an ideal implementation—our implementation may scale worse than this depending on the efficiency of the underlying communication logic. Note that the number of distinct event times *J* is bounded by the number of subjects *n*. In our experiments, they are of the same order of magnitude

The preprocessing consists out of a matrix-matrix product for sorting of the secret-shared data ($n^2$ invocations), where we leverage the efficient inner products in MPyC, and a pre-computation of all $np(p+1)/2$ cross-products of covariates ($Z_r^I Z_s^I$). The significant contribution of the secure exponentiation protocol is mainly caused by the fact that it is performed for $n(n-1)/2$ exponents per iteration. The number of invocations and communication rounds grows linearly in the precision parameter $\tilde{k}$.

The gradient and Hessian are computed from the $\theta ^k$ [Eq. (10)]. Since Eq. (10) is recursive, this requires the computation of *n* secure reciprocals in parallel. The Hessian matrix can then be computed by performing $O(Jp(p+1)/2)$ secure multiplications in parallel. Subsequently, the Hessian matrix needs to be inverted. This subprotocol requires two conversions between secure types in the MPyC library: from SecFxp to SecFld and vice versa. The conversion protocol is dominated by a secure modular reduction, which scales linearly in the bit-length of the modulus of the enlarged secure field: $O(p\log p)$ invocations and $O(\log p)$ rounds. Converting all elements of the matrix in parallel results in the stated complexity.

Finally, $\beta$ is updated by performing a matrix-vector multiplication after which the convergence criterion is verified at the cost of *O*(*p*) secure comparisons.

In conclusion, if *R* is the number of Newton–Raphson iterations, then the theoretical performance is dominated by $O(R(\tilde{k} n^2 + np^2 + p^3\log p))$ invocations in $O(R(n + \log p))$ rounds. Note that MPyC is not yet optimized for reducing the number of communication rounds and it is very well possible that the current implementation of the secure CPH protocol initiates many more rounds than optimal.

## Results and discussion

To gain insights in the practical scalability of the implemented protocol, we ran several experiments. The main purpose of these experiments was to determine the accuracy, complexity and scalability of the implementation. Naturally, the accuracy of the privacy-preserving CPH implementation should be close to that of the baseline implementations in order to be useful. However, the privacy-preserving implementation is significantly more demanding in terms of computational power, storage and network communication and therefore the relative performance of the protocol should be tested as well. In particular, we wonder how the training times grow if the input data sets increase in size (considering both the number of covariates as well as the number of records)? We remark that the gathered training times unavoidably depend on the power of the CPUs being used; the absolute data points should therefore be solely used to obtain an intuition on the performance of the (sub)protocols.

### MPC platform

Our solution can be implemented in any linear secret sharing platform. We implemented the solution using the MPyC [11] platform, which bases its protocols on Shamir secret sharing [12] and pseudorandom secret sharing. A benefit of the MPyC platform facilitates asynchronous evaluation of MPC protocols, implying that a party can perform local computations (e.g. generate randomness) while she is waiting for other players’ information. Another main reason for using MPyC is its communication-efficient protocol for performing inner products, which is a key operation in our protocol for training the CPH model.

Shamir secret sharing and therefore MPyC requires at least $m\ge 3$ computing parties for security reasons. As such, if there are in fact only two parties that provide data for training the CPH model, we assume that a semi-trusted third party (helper) joins the computation such that we meet the security requirements. The helper is not allowed to learn any sensitive values, including model parameters and explanatory covariates. In fact, the helper does not even need to learn the final outcome of the model. We do assume that the helper is semi-honest and that he does not collude with other parties.

### Security analysis

Let’s assume two parties aim to jointly compute a secure CPH on their vertically-partitioned data. For example a study aimed at measuring the impact of drugs registered by an insurance agency with respect to the vital status and follow-up time that is recorded in a disease specific registry. As in any MPC platform, we assume that each pair of parties has an authenticated and confidential communication channel to securely exchange messages. This avoids eavesdroppers to learn any sensitive information on the parties’ inputs.

The MPyC framework assumes that (the consortium of at least three) parties are honest-but-curious and that they do not collude. This means that every party will adhere to the protocol and might only try to learn from the information that it has received during the execution of the protocol. The information that is received is not supposed to be shared with other parties (colluding). If a stronger security model, e.g. with cheater detection, or a scenario that allows for just two parties, e.g. without semi-trusted helper party, is desired, then another framework must be used and the protocols described in this document will have to be re-evaluated. However, in most application scenarios this framework is considered to be adequate, as each respective data holder is putting their own data and reputation on the line, which would make it unlikely for them to deviate from the protocol.

Our protocols are built from default computation steps that have been implemented within the MPyC platform, and therefore inherit its security properties. The only exceptions are our new secure exponentiation protocol, and the matrix inverse protocol, which both reveal intermediate values. The first one reveals $\tilde{y}=-y+\sum _{i=1}^m r_i$ (see step 3 of Protocol 3), where the sensitive value *y* is statistically blinded by the $r_i$, because they have $\sigma$ more bits than *y*. The second one reveals the matrix *RA* (see step 2 of Protocol 5), which has been proven secure in [31].

### Experiment

The experimental data were gathered in a distributed setting. Three machines were installed on three different (geographical) locations, and the interactive protocol was executed with reliable, high-throughput communication channels over the internet. All parties had a similar set-up, the implementation was ran within a Docker environment on Ubuntu 18.04 LTS. Every machine was equipped with 16 GBs of RAM and four virtual cores.

#### Accuracy

We ran experiments to validate the accuracy of our secure solution for training the CPH model. We used three data sets and benchmarked those on three different implementations:

- Built-in R implementation [32] (also refered to as ‘lib’);
- Plaintext Newton R implementation (also refered to as ‘newton’), and;
- Secure Newton implementation (also refered to as ‘mpc’).

Note that the built-in R implementation uses an alternative, more optimized (but also more complex) solver resulting in a slightly modified model. It is therefore quite possible that this optimizer converges in less iterations. Furthermore, it uses the Efron approximation as a default for handling tied event times, which we set to Breslow to properly compare performance. To fairly compare, we also implemented a plaintext variant that uses the Newton–Raphson method for optimizing Breslow’s partial likelihood function. This proves useful as a benchmark to compare the loss of accuracy with the needed number of iterations before passing the set threshold.

The reasoning behind benchmarking three implementations is the following. Differences in accuracy between ‘lib’ and ‘newton’ are caused by the different solvers. Typically, this shows that the Newton–Raphson solver that we based our protocol on is a decent solver, but the method itself is just not optimal. The secure ‘mpc’ implementation should ideally have identical performance to ‘newton’ as it is based on the same solver. However, both the fact that ‘mpc’ uses fixed-point representations and that several approximations were made in the secure implementation imply that differences may occur. Comparing ‘newton’ and ‘mpc’ gives an experimental indication of the accuracy impact caused by making the solver secure.

The analysis was performed on the following default lifelines survival datasets [33] after filtering for missing values:

- Larynx (90 patients, four covariates);
- Leukemia (42 patients, three covariates), and;
- Lung (167 patients, eight covariates).

The covariates are distributed among the parties (party 1 and 2) as given in Table 3. We set the convergence criterion to $2^{-11}\approx 4.8828 \times 10^{-4}$ for all implementations. The results are presented in Tables 4, 5, and 6. .

**Table 3:** Vertical partitioning of covariates per party per dataset

Dataset	Covariates party 1	Covariates party 2
Larynx	Age	Stage_II, Stage_III, Stage_IV
Leukemia	Sex	LogBC, Rx
Lung	Inst, age	Sex, ph.ecog, ph.karno, pat.karno, meal.cal, wt.loss

**Table 4:** Larynx dataset. Coefficients (coef) and standard error (se) are listed for each implementation

Covariates	coef_lib	coef_newton	coef_mpc	se_lib	se_newton	se_mpc
Age	0.018900	0.018902	0.018906	0.014251	0.014251	0.014228
Stage_II	0.138424	0.138564	0.138550	0.462319	0.462319	0.462293
Stage_III	0.638148	0.638350	0.638260	0.356090	0.356090	0.356112
Stage_IV	1.693331	1.693056	1.692993	0.422179	0.422179	0.422164

Convergence was reached in three iterations for ‘lib’, and in four iterations for ‘newton’ and ‘mpc’. The secure implementation ‘mpc’ took 740 seconds to complete

**Table 5:** Leukemia dataset. Coefficients (coef) and standard error (se) are listed for each implementation

Covariates	coef_lib	coef_newton	coef_mpc	se_lib	se_newton	se_mpc
Sex	0.263177	0.263171	0.263107	0.449435	0.449435	0.449439
logWBC	1.593608	1.593619	1.593384	0.329995	0.329995	0.329993
Rx	1.390869	1.390877	1.390930	0.456645	0.456645	0.456630

Convergence was reached in three iterations for ‘lib’, and in four iterations for ‘newton’ and ‘mpc’. The secure implementation ‘mpc’ took 167 seconds to complete

**Table 6:** Lung dataset

Covariates	coef_lib	coef_newton	coef_mpc	se_lib	se_newton	se_mpc
Inst	$-$0.011852	$-$0.011861	$-$0.011856	0.010921	0.010921	0.010930
Age	0.000026	0.000027	$-$0.000046	0.009779	0.009779	0.009848
Sex	$-$0.251135	$-$0.251185	$-$0.251434	0.163212	0.163212	0.163282
ph.ecog	0.615030	0.614995	0.615158	0.204500	0.204500	0.204551
ph.karno	0.023395	0.023392	0.023376	0.010189	0.010189	0.010203
pat.karno	$-$0.009492	$-$0.009487	$-$0.009491	0.007027	0.007027	0.007047
meal.cal	$-$0.000080	$-$0.000080	$-$0.000076	0.000227	0.000227	0.000227
wt.loss	$-$0.011039	$-$0.011043	$-$0.011063	0.006606	0.006606	0.006607

Coefficients (coef) and standard error (se) are listed for each implementation. Convergence was reached in two iterations for ‘lib’, in three iterations for ‘newton’ and ‘mpc’. The secure implementation ‘mpc’ took 3073 seconds to complete

For all three datasets, the computed coefficients in the secure version (‘coef_mpc’) are close to their plain text variants (‘coef_newton’), and the latter are close to the ones computed with built-in R solvers (‘coef_lib’). The same assertion holds for the computed standard errors, indispensable to calculate *p*-values and confidence intervals for the estimated coefficients. Note that, the proposed MPC approach provides *p*-values that are approximated up to the fourth digits and therefore sufficient to derive the same statistical conclusions on the study. We conclude that the secure implementation achieves a much higher level of confidentiality with very little impact on accuracy. The difference in terms of iterations between the built-in R solver and our less optimized, and less complex, solver is just one. This acceptable difference in convergence is due to the fact that we do not optimize the step-halving procedure.

#### System performance

We now evaluate the time needed for the joint servers to execute the subprotocols and full protocol. The execution time of a protocol is an aggregate of computation time (the server is performing computations) and communication time (the server is sending, receiving or waiting for messages from other players). We do not distinguish between these two for two reasons. First, the MPyC framework that we use executes the protocols asynchronously. This renders distinguishing between and monitoring of different modes of operation very delicate and the framework provides no support for doing so. Second, we also ran local experiments on a single server that simulated a multi-party execution of the protocol. The results of these experiments were indistinguishable from the final, distributed set-up. This observation suggests that, given the network conditions during our distributed experiments, the communication cost in our distributed set-up is negligible compared to the computation cost.

**Fig. 1:**
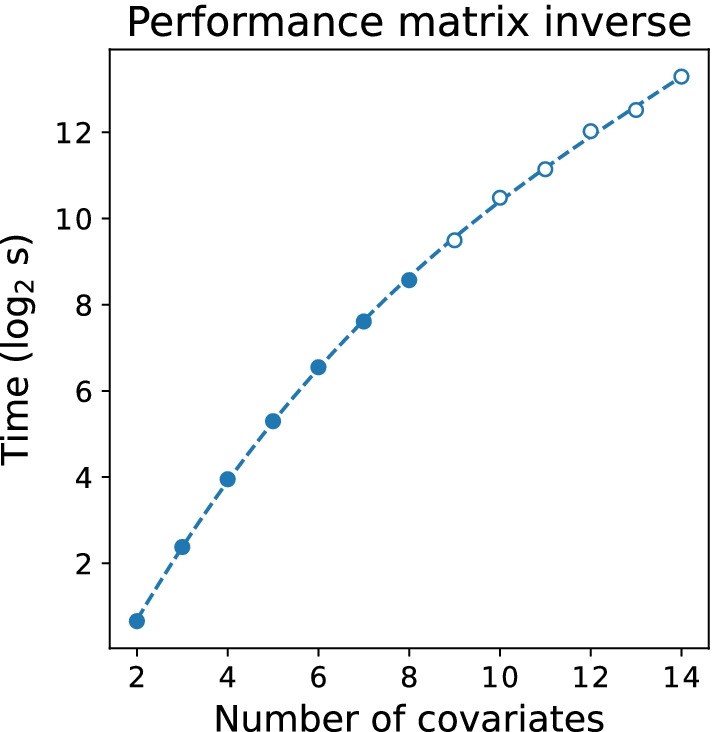
Performance of the matrix inverse protocol. This figures demonstrates the scalability of the matrix inverse in the number of covariates (dimension of the matrix). The filled data points are based on an average of 100 runs per datapoint. The open data points are based on a single run. We need to perform one matrix inversion per iteration of the secure CPH protocol. Remark: the current implementation supports matrix inversions of matrix sizes of $2\times 2$ upto $14\times 14$

**Fig. 2:**
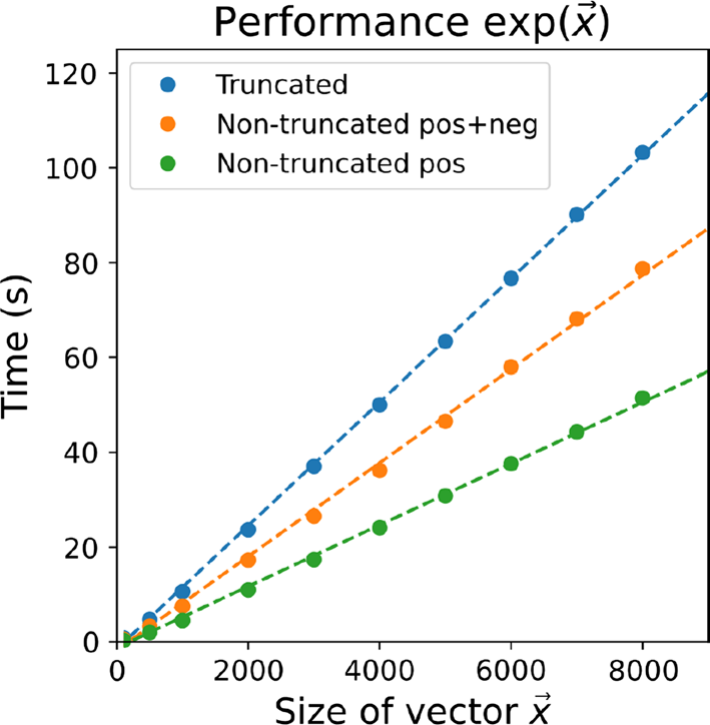
Performance of the exponentiation protocol. The data points are based on an average of 100 runs per datapoint. We observe a linear scaling in the size of the vector *x*. Remark: we need $n(n-1)/2$ invocations of the exponentiation, where *n* is the number of sample (or patients), resulting in quadratic scaling in the number of samples. A more elaborate explanation of the legend; Green: exponents are assumed to be in the interval [0, 12]. No truncation is performed to enforce this, resulting in zero secure comparisons to perform the exponentiation; Orange: exponents are assumed to be in the interval $[-12, 12]$. No truncation is performed to enforce this, however one secure comparison needed to deal with negative exponents; Blue: given an interval $[-x, y]$ (e.g., $[-12, 12]$), all exponents are truncated to fit in this range to prevent overflows. Two secure comparisons are needed to achieve this

**Fig. 3:**
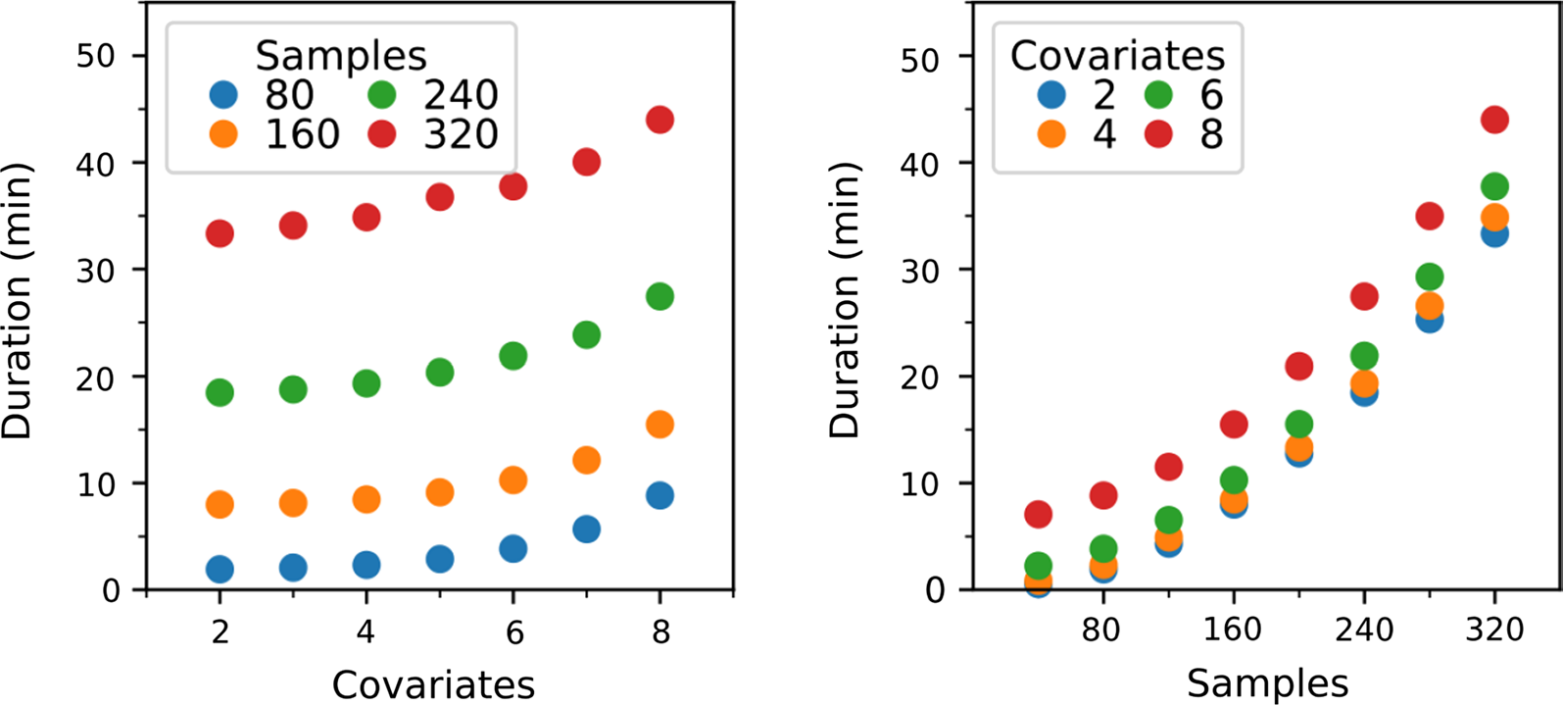
Performance of the overall Cox proportional hazards protocol, experimental data was gathered by performing a single run per data point. The number of iterations per run was fixed to five. The visualized duration is given in minutes per iteration

To benchmark the system performance of the secure implementation, we first elaborate upon the system performance of the matrix inverse and the exponentiation protocols, visualized in Figs. 1 and 2 respectively, as these protocols dominate the costs of running the overall protocol. The performance of the entire secure CPH protocol is displayed in Fig. 3.

Analyzing Fig. 1, we observe a performance in line with the theoretical analysis. We remark that the current implementation only supports matrix inversions of matrix sizes of $2\times 2$ upto $14\times 14$, due to limitations within MPyC and Python that limit the maximum size of a floating point.

Figure 2 demonstrates the practical performance of the implemented secure exponentiation. It is shown that it scales linearly in the number of inputs for various variants of the protocol explained in “Wrapper for truncation” section. We observe that the computational complexity of the non-truncated positive variant is mainly due to the Taylor series approximation involved in the secure exponentiation. That part computes several powers of some term in series (e.g., eight-term approximation requires six secure multiplications in series). The added complexity for the other variants are due to the additional secure comparisons.

The overall experimental performance of our implementation of the Cox proportional hazards protocol is illustrated in Fig. 3.

For these experiments, the convergence criterion was disabled and the number of iterations was fixed to five for consistency. Note that the secure iterative algorithm needed at most four iterations to convergence in our experiments with the lifelines survival datasets. The number of covariates in our experiments is limited due to the matrix inverse that was discussed before; as such, we cannot make any rigorous statements about the experimental scaling properties in that dimension. Alternatively, the impact of the number of samples on the computation time reflects the quadratic scaling that we deduced in the complexity analysis.

### Discussion

A comprehensive clinical study generally benefits from combining patient data from different sources [34, 35]. For example, when linking insurance data with a cancer registry, the progression of cancer can be monitored and reveal how certain treatments can lead to different chances of survival.

In this paper we propose a secure version of the CPH model. It is one of the most important statistical techniques in medical research for investigating the association between patient survival time and one or more explanatory variables. While CPH can show how treatments are associated with survival, it does not explain what causes the direction of the association. Positive or negative associations might be explained by treatment itself, the types of patients the treatment is performed on, the progression of the disease, or other factors. Hence, conclusions drawn from a CPH model always require further scrutiny. This is a common challenge in research on observational data, but becomes even more important when this data is collected by different parties. Before the study commences, all parties need to have sufficient understanding of each other’s data collection process. Additionally, exploratory analysis and input validation tests are needed to rule out any other factors that could explain differences in survival.

While the techniques discussed in the paper are promising, actual usage in a real-world scenario can prove to be quite challenging. Before a joint study commences, all parties require legal consent and approval to perform studies on their (securely) combined data sources. This generally requires elaborate discussions between the participating organisations with input from different disciplines, including legal, management, researchers and software developers. Sufficient resources need to be allocated for having these multilateral, multidisciplinary discussions as this process cannot and should not be overstepped.

Still, MPC is an attractive alternative to traditional data linkages, where the latter generally has much higher risks of privacy breaches [36]. However, MPC algorithms are generally still subjected to inherent technical challenges, particularly with respect to large computational complexity. This is also the case for the algorithm presented in this paper. The computational burdens of our approach limits its usage to clinical studies that involve relatively small sample sizes (such as used in [37–39]), however it represents an important starting point for future research on the development MPC algorithms.

#### Future work

The accuracy of the solution (and possibly the scalability) of the protocol through the size of the secret-sharing modulus suffers from the limitation of MPyC to fixed-point arithmetic. Once floating-point arithmetic becomes available in the MPyC framework, we might be able to significantly improve the accuracy (and scalability) of the solution. Furthermore, floating-point arithmetic will greatly improve memory usage of the matrix inverse subprotocol, we expect that the maximal dimension of the matrix to invert is no longer bounded by $14\times 14$.

We can greatly reduce the computation time of the overall protocol by parallelizing the exponentiations that need to be performed in every iteration. Parallelization is currently not supported in the MPyC framework. Low-hanging fruit would be to only parallelize the generation of randomness needed in the exponentiation (sub)Protocol 3. An alternative could be to precompute this randomness before execution of the protocol.

Finally, it would be interesting to investigate solutions with slightly different accuracy-efficiency-privacy trade-offs. For example, one could be interested in a more efficient and scalable matrix inversion protocol even if it provides weaker privacy guarantees.

## Conclusions

A secure version of the Cox proportional hazards model enables researchers to study survival probabilities of patients while taking into account covariates over distributed databases. Data from multiple institutions no longer has to be shared or combined to perform a comprehensive study of patient survival. This provides strong protection of patient data while enabling novel forms of research. Our secure version of the CPH allows for more information to be included in clinical studies, which potentially can lead to new insights on which factors impact the survival of patients.

The secure solution is comparable with the plaintext solver in terms of accuracy and convergence speed. The computation time is considerably larger, however the theoretical complexity is still cubic in the number of covariates, and quadratic in the number of subjects.

In conclusion, the solution in this paper can enable organisations to safely perform parametric survival analysis on vertically-distributed medical data, while guaranteeing a high level of security and privacy.

## Supplementary information

**Additional file 1**: Exponentiation approximation. It describes the details of the MacLaurin series approximation in “Non-integer exponent” section

**Additional file 2**: Matrix inversion background. It briefly introduces core components of the secure matrix inversion protocol by Blom et al. [31], as referred to in “Matrix inverse protocol” section.

## Data Availability

The datasets generated and/or analysed during the current study are available in the Lifelines repository [33], https://github.com/CamDavidsonPilon/lifelines/tree/97c455d13cf2aaba5b99abd6b01476ce4415b6d3/lifelines/datasets.

## Footnotes

1 https://gdpr-info.eu.

2 https://www.govinfo.gov/content/pkg/PLAW-104publ191/pdf/PLAW-104publ191.pdf.

3 Source code of VERTICOX is available at https://github.com/daiwenrui/VERTICOX.
