# Supplementary material for: Accurate training of the Cox proportional hazards model on vertically-partitioned data while preserving privacy
Source: BMC Med Inform Decis Mak. 2022 Feb 24;22:49. doi: 10.1186/s12911-022-01771-3 (PMC8867891; doi:10.1186/s12911-022-01771-3)
Supplement: Supplementary file 1 — Additional file 1: Exponentiation approximation. It describes the details of the MacLaurin series approximation in “Non-integer exponent” section [file 12911_2022_1771_MOESM1_ESM.pdf]

Additional file 1
Exponentiation approximation

Bart Kamphorst, Thomas Rooijakkers, Thijs Veugen, Matteo Cellamare, Daan Knoors

This additional file describes the details of the MacLaurin series approximation in section “Secure exponentiation protocol”, subsection “Non-integer exponent”. The MacLaurin series of e^s can, for any $\tilde{k} \geq 0$, be represented as:

$$e^s = \sum_{k=0}^{\infty} \frac{s^k}{k!} = \sum_{k=0}^{\tilde{k}} \frac{s^k}{k!} + \sum_{k=\tilde{k}+1}^{\infty} \frac{s^k}{k!}. \tag{1}$$

From this expression, we find an upper for the absolute difference between e^s and the finite sum:

$$\begin{aligned} 0 \leq \left| e^s - \sum_{k=0}^{\tilde{k}} \frac{s^k}{k!} \right| &= \left| \sum_{k=\tilde{k}+1}^{\infty} \frac{s^k}{k!} \right| \\ &\leq \frac{|s|^{\tilde{k}+1}}{(\tilde{k}+1)!} \left| \sum_{k=\tilde{k}+1}^{\infty} \frac{s^{k-\tilde{k}-1}}{(k-\tilde{k}-1)!} \right| = \frac{|s|^{\tilde{k}+1}}{(\tilde{k}+1)!} |e^s|. \end{aligned} \tag{2}$$

We now substitute $s = z\log(a)$, $a > 0$, to find

$$0 \leq \left| a^z - \sum_{k=0}^{\tilde{k}} \frac{(z\log(a))^k}{k!} \right| \leq \frac{|z\log(a)|^{\tilde{k}+1}}{(\tilde{k}+1)!} |a^z|. \tag{3}$$

For a given base a and a given range $[-Z, Z]$ that contains z, the relative error of the approximation can be made arbitrarily small by choosing suitably large $\tilde{k}$. To see this, recall that the factorial function grows faster than any function that grows polynomially (in the logarithm of the argument).
