# Supplementary material for: Accurate training of the Cox proportional hazards model on vertically-partitioned data while preserving privacy
Source: BMC Med Inform Decis Mak. 2022 Feb 24;22:49. doi: 10.1186/s12911-022-01771-3 (PMC8867891; doi:10.1186/s12911-022-01771-3)
Supplement: Supplementary file 2 — Additional file 2: Matrix inversion background. It briefly introduces core components of the secure matrix inversion protocol by Blom et al. [31], as referred to in “Matrix inverse protocol” section. [file 12911_2022_1771_MOESM2_ESM.pdf]

# Additional file 2
# Matrix inverse background

Bart Kamphorst, Thomas Rooijakkers, Thijs Veugen, Matteo Cellamare, Daan Knoors

This additional file very briefly introduces core components of the secure matrix inversion protocol by Blom et al. [1], as referred to in section "Matrix inverse protocol".

*Random matrix with determinant*

To perform the first step, a random $LU$ decomposition is generated. Such matrices are statistically undistinguishable from uniformly random matrices [1].

1 The parties generate a random lower triangular (encrypted) matrix $L$ with ones on the diagonal (such that $\det L = 1$).
2 The parties generate a random upper triangular (encrypted) matrix $U$, and securely compute the reciprocal $(\det U)^{-1} = (\prod_{i=1}^{d} u_{i,i})^{-1}$.
3 They compute $[R] = [L] \cdot [U]$ with a secure matrix product.

Since $\det L = 1$, the reciprocal of the determinant $(\det R)^{-1}$ will be equal to the reciprocal $(\det U)^{-1}$, which we securely compute from $[\det U]$ as follows:

1 The parties generate an encrypted, uniformly random number $[r]$.
2 They securely compute $[r \det U]$, decrypt the result and compute $(r \det U)^{-1}$
3 They locally multiply the result with $[r]$ to obtain $[(\det U)^{-1}]$

In the unlikely case that $r \det U = 0$, we have generated a singular matrix $U$, and need to regenerate it.

*Gauss-Jordan elimination*

With Gaussian elimination, the first part of the augmented matrix can be transformed to the identity matrix. It will take a sequence of three elementary row operations, namely swapping of two rows, multiplying (or dividing) a row with an integer factor, and adding two rows. As a bonus, the Gaussian elimination facilitates computing the determinant of $\vec{RA}$: each row multiplication divides the determinant with that scalar, and each swap negates the determinant.

The first step in Gaussian elimination is transforming $\vec{RA}$ to an upper triangular matrix. This can done without divisions, and adding only multiplications of rows. The second step is transforming the upper triangular matrix to a diagonal matrix, which is performed similarly [2]. Only the third and final step, transforming the diagonal matrix to the identity matrix, involves divisions, which comes down to $d$ multiplications with multiplicative inverses.

**References**

1. Blom, F., Bouman, N., Schoenmakers, B., Vreede, N.: Efficient Secure Ridge Regression from Randomized Gaussian Elimination. IACR Cryptol. ePrint Arch. (2019)
2. Bareiss, E.H.: Sylvester's Identity and Multistep Integer- Preserving Gaussian Elimination. Math. Comp. **22**, 565–578 (1968). doi:10.1090/S0025-5718-1968-0226829-0
